# A High-Throughput Model-Assisted Method for Phenotyping Maize Green Leaf Area Index Dynamics Using Unmanned Aerial Vehicle Imagery

**DOI:** 10.3389/fpls.2019.00685

**Published:** 2019-06-06

**Authors:** Justin Blancon, Dan Dutartre, Marie-Hélène Tixier, Marie Weiss, Alexis Comar, Sébastien Praud, Frédéric Baret

**Affiliations:** ^1^Biogemma, Centre de Recherche de Chappes, Chappes, France; ^2^HIPHEN SAS, Avignon, France; ^3^INRA UMR 114 EMMAH, UMT CAPTE, Domaine Saint-Paul, Avignon, France

**Keywords:** diversity panel, dynamics, drought, green leaf area index (GLAI), growth model, high-throughput phenotyping, maize, unmanned aerial vehicle (UAV)

## Abstract

The dynamics of the Green Leaf Area Index (GLAI) is of great interest for numerous applications such as yield prediction and plant breeding. We present a high-throughput model-assisted method for characterizing GLAI dynamics in maize (*Zea mays* subsp. *mays*) using multispectral imagery acquired from an Unmanned Aerial Vehicle (UAV). Two trials were conducted with a high diversity panel of 400 lines under well-watered and water-deficient treatments in 2016 and 2017. For each UAV flight, we first derived GLAI estimates from empirical relationships between the multispectral reflectance and ground level measurements of GLAI achieved over a small sample of microplots. We then fitted a simple but physiologically sound GLAI dynamics model over the GLAI values estimated previously. Results show that GLAI dynamics was estimated accurately throughout the cycle (R^2^ > 0.9). Two parameters of the model, biggest leaf area and leaf longevity, were also estimated successfully. We showed that GLAI dynamics and the parameters of the fitted model are highly heritable (0.65 ≤ H^2^ ≤ 0.98), responsive to environmental conditions, and linked to yield and drought tolerance. This method, combining growth modeling, UAV imagery and simple non-destructive field measurements, provides new high-throughput tools for understanding the adaptation of GLAI dynamics and its interaction with the environment. GLAI dynamics is also a promising trait for crop breeding, and paves the way for future genetic studies.

## Introduction

Crop production is mainly driven by the plant's capacity to intercept and use sunlight through photosynthesis. Photosynthetically active radiation is mostly intercepted by leaves, which are also the principal interface for water and carbon exchanges. However, green leaf area is influenced by several stresses including nitrogen, water and temperature (Çakir, 2004; Ding et al., 2005; Chen et al., 2018), thus reducing dry matter production and yield. This underlines the importance of green leaf area estimation for several applications such as yield prediction (Baez-Gonzalez et al., 2005; Dente et al., 2008), precision farming (Walthall et al., 2007), and plant breeding (Yang et al., 2017b). Green leaf area can be quantified by the Green Leaf Area Index (GLAI) defined as the one-sided green area of leaves per unit horizontal ground surface area (Chen and Black, 1992). Its dynamics throughout the crop cycle is considered as a crucial trait for improving grain yield and adapting a genotype to a particular environment and climatic scenario (Bänziger et al., 2000; Tardieu, 2012).

Different approaches have been developed to estimate GLAI including *in-situ* methods, remote sensing techniques, and crop models. Direct measurements of the area of a sample of green leaves in the canopy is time-consuming, labor-intensive and prone to errors when the sampling size is too small. Indirect *in-situ* methods based on light transmission through the canopy (Jonckheere et al., 2004) are easier to implement than direct GLAI measurements. However, both these direct and indirect ground-based methods remain tedious and low-throughput, thus failing to satisfy breeders' requirements which entail the characterization of hundreds to thousands of microplots several times throughout the growth cycle. Therefore, high-throughput methods for estimating GLAI are highly desirable. Remote sensing observations from UAVs (Unmanned Aerial Vehicles) present the advantage of fulfilling spatial and temporal resolution requirements while providing high-throughput measurements at a relatively low cost, making it a valuable phenotyping tool (Tattaris et al., 2016; Yang et al., 2017a; Reynolds et al., 2018).

Remote sensing methods rely on the use of multispectral or hyperspectral sensors to measure canopy reflectance, which is sensitive to variation in the GLAI. Empirical methods have been widely used to statistically relate the GLAI to the reflectance observed in several bands generally combined in vegetation indices. The results of this approach depend on the sensitivity of the selected vegetation indices to the GLAI but also to confounding factors such as leaf orientation, illumination conditions and soil properties (Baret and Guyot, 1991). Moreover, this approach must be applied in the same conditions as those prevailing during the calibration of the statistical relation, as it lacks robustness and accuracy when applied under other conditions, i.e., outside the calibration domain (Broge and Leblanc, 2001; Haboudane et al., 2004; Dorigo et al., 2007). More comprehensive statistical models, that are more robust and can be applied to different crops, were also developed (Viña et al., 2011; Nguy-Robertson et al., 2014; Kang et al., 2016; Kira et al., 2017). However, empirical transfer functions are generally calibrated and applied locally, thus limiting possible extrapolation problems. They thus must be calibrated each time over a set of samples representative of the range of variation.

The dynamics of GLAI is of prime importance to understand the functioning of crops. The growth and senescence rates of the leaf area, the timing of the minimum and maximum GLAI and the corresponding magnitude are important traits for breeders (Comar et al., 2012). The continuous description of GLAI dynamics based on crop models like APSIM (Keating et al., 2003), STICS (Brisson et al., 1998), and DSSAT (Jones et al., 2003) would provide a very efficient solution to access similar functional traits, corresponding to genotype-dependent parameters. However, the complexity of such models, the large number of parameters required and the mandatory information on important environmental conditions that are often not well-known, still make it difficult to broaden their use (Liu et al., 2014; Gaydon et al., 2017). Nevertheless, simpler semi-empirical models that require a minimum set of parameters with physiological meaning and a limited description of the environment have already been used to estimate GLAI dynamics or to interpolate and smooth remote sensing observations collected throughout the cycle (España, 1997; Kötz, 2001; Lizaso et al., 2003). Such simple dynamic models therefore appear well-adapted in situations where information on environmental conditions is limited and when only few field measurements are possible.

The objectives of this study are (i) to propose a high-throughput phenotyping method to describe maize (*Zea mays* subsp. *mays*) GLAI dynamics from UAV observations repeated throughout the growth cycle and a simple but physiologically sound GLAI dynamics model (ii) to unravel GLAI dynamics response to environmental scenarios, and (iii) to investigate the potential interest of GLAI traits for maize breeding in well-watered and water limited environments.

## Materials and Methods

A simple model inspired from the work of Baret (1986), España (1997), Kötz (2001), and Lizaso et al. (2003) is proposed to simulate GLAI dynamics from a limited set of parameters. Empirical transfer functions are first calibrated for each flight to estimate the GLAI from the UAV observations and additional available predictors. The simple GLAI dynamics model is then fitted to the GLAI estimates from the transfer functions by adjusting the unknown parameters. The heritability of the derived traits describing the GLAI dynamics is quantified. Their effect on grain yield is evaluated as well as the genotypic response to water stress.

### Plant Material, Experimental Design and Environmental Conditions

The study was carried out on a panel of lines derived from a MAGIC population (Multi-parent Advanced Generation Inter-Crosses). This population was created following a funnel crossing design from 16 historical lines representative of the genetic diversity of temperate material. The panel consisted of 400 doubled haploid lines extracted from the third generation of population mixing (Buet et al., 2013). The doubled haploid lines were crossed with the tester line MBS847 and their progenies evaluated in the field. Phenotypic evaluations from test-cross progenies aimed at comparing lines in a hybrid context and reducing the range in flowering time to limit confounding effects due to differences in precocity.

Field trials were conducted in 2016 and 2017 close to Romans-sur-Isère, France (45° 4′N, 5° 6′E) with, respectively, 360 and 347 hybrids (330 in common). Each year, two trials were carried out, one in a Well-Watered (WW, irrigated) condition and the other in a Water-Deficient (WD, rain-fed with monitored irrigation) condition. The experiment was laid out as an alpha-lattice design with two replicate blocks for each treatment in both years. The plot length was 5.35 m with 2 rows spaced by a 0.8 m interval. The soil water potential was measured at three different depths (30, 60, and 90 cm) with a tensiometer in each treatment throughout the cycle. Because the flowering time is known to be the most drought sensitive period for grain yield, the WD trial irrigation was monitored to target a water deficit from 10 days before flowering time to 10 days afterwards. Moreover, this timing was expected to impact not only the end of leaf development and thus the GLAI amplitude, but also leaf longevity. The trials were sown on 6^th^ May 2016 and on 18^th^ May 2017 at a density of 9 seed.m^−2^ in a sandy loam soil. The silking stage (defined as the time when 50% of a plot has visible silks) was reached around 19^th^ July (≈950°C.d) in 2016 and around 16^th^ July (≈900°C.d) in 2017. Weeds, diseases and pests were controlled using conventional agronomic practices.

### GLAI Dynamics Maize Model (GDMM)

The model of maize GLAI dynamics was derived from the previous models proposed by Baret (1986), España (1997), Kötz (2001), and Lizaso et al. (2003). Time is described by growing degree days (GDD) computed using a 6°C base (Sánchez et al., 2014). Growing degree days control the rate of leaf appearance using the phyllochron ϕ_*GDD*_, i.e., the GDD required between the appearance of two successive leaves. A leaf is considered appeared when its tip emerges visibly out of the whorl. To account for the quick appearance of the first four leaves, the phyllochron is set to $\phi_{GDD}=20{}^{\circ}C.d$. After leaf emergence, leaf area expands linearly with GDD until the leaf area expansion is completed, when the ligule emerges from the whorl (Supplementary Figure 1). The leaf stage when the ligule of the leaf *i* appears, *L*_*i*_, is estimated as:

(1) $$\begin{array}{l} L_{i}=4.55+0.76\;i_{top}-1.06\frac{{\left(i-i_{top}\right)}^{2}}{i_{top}} \end{array}$$

where *i*_*top*_ is the final number of leaves.

*L*_*i*_ and ϕ_*GDD*_ are then used to compute the GDD at which the ligule appears.

The Maximum Area of leaf *i, MA*_*i*_, reached when the ligule appears, is computed as:

(2) $$MA_{i}=\frac{1+\sin\left(\frac{\pi }{0.5926}\times {\left(\frac{i}{i_{top}}\right)}^{1.4158}-\frac{\pi }{2}\right)}{2}\times MA_{big}$$

where *MA*_*big*_ is the maximum area of the biggest leaf.

Leaf longevity, δ_*i*_, defined as the GDD required between leaf appearance and leaf death, depends on leaf order i:

(3) $$\begin{array}{l} \delta_{i}=\delta \left[\frac{1}{1+e^{-0.77\left(i-\frac{i_{top}}{3}\right)}}-e^{0.16\left(i-\left(i_{top}+3.5\right)\right)}\right]+550 \end{array}$$

**Equation 3** is an adaptation of the exponential models proposed by Baret (1986) to describe LAI dynamics and that of Lizaso et al. (2003) describing leaf senescence. The start of senescence for each leaf is set equal to 75% of leaf longevity, δ_*i*_. Senescence is assumed to be linear with GDD from its start up to the death of the leaf (Supplementary Figure 1).

The model proposed therefore describes the dynamics of the green leaf area of each plant as the sum of the green area of each individual leaf. GLAI is finally computed by multiplying the plant green leaf area by the plant density, *d*. The resulting GDMM uses 5 parameters: {*d, i*_*top*_, ϕ_*GDD*_, *MA*_*big*_, δ} (Table 1). Every parameter has a specific effect on the GLAI dynamics, except *d* and *MA*_*big*_ that have a similar impact (Supplementary Figure 2).

**Table 1:** Parameters required for the GLAI Dynamics Maize Model (GDMM) and their ground measurement in this study.

**Parameter**	**Description**	**Unit**	**Ground measurements**
*d*	Plant density	plant.m^−2^	All microplots
*i*_*top*_	Final leaf number	leaf	Well-Watered 2016
ϕ_*GDD*_	Phyllochron	°C.d	Well-Watered 2016
*MA*_*big*_	Maximum area of the biggest leaf	m^2^	Reference microplots
δ	Leaf longevity factor	°C.d	Reference microplots

### Ground Measurements

#### Measurements Performed Over the Whole Experiment

The appeared leaves were counted on a weekly basis on three plants identified on each of the microplots in one replicate of the WW treatment in 2016. This provided an estimate of the phyllochron, ϕ_*GDD*_, as well as the final number of leaves, *i*_*top*_. These parameters have been shown to be dependent on the genotype and stable between environments (Hajibabaee et al., 2012; Millet, 2016; Parent et al., 2018). They were thus assumed to be dependent only on the genotype and measured in 2016 over the WW treatment. These values of {*i*_*top*_, ϕ_*GDD*_} were used for the WD treatment in 2016 and for both treatments in 2017.

The flowering date (silking date in °C.d) was recorded for each microplot, as well as the plant density, *d*, at maturity. Plants were then harvested on 10^th^ October 2016 (≈2,100°C.d) and on 25^th^ October 2017 (≈2,150°C.d) to estimate grain yield adjusted to 15% moisture (in q.ha^−1^), thousand kernel weight adjusted to 15% moisture (in g), kernel number per square meter and harvest grain moisture (in %).

#### GLAI Ground Measurements Over a Reference Sample of Microplots (GLAI_field_)

In 2016 and 2017, for both conditions (WW and WD) and the two replicates, a reference sample of 15 (2016) and 20 (2017) genotypes was selected amongst which 10 were common between years. This resulted in 15 genotypes × 2 replicates × 2 water regimes = 60 microplots in 2016 and 20 genotypes × 2 replicates × 2 water regimes = 80 microplots in 2017. Genotypes were chosen to be contrasted for both GLAI magnitude and dynamics.

Shortly after flowering, the width, *w*, and length, *l*, of the biggest leaf were measured on three plants per microplots and the corresponding area estimated as *MA*_*big*_ = 0.72.*w*.*l* (España, 1997). **Equation 2** was then used to estimate the area of fully expanded leaves. The fraction of green area of each leaf was visually scored on a weekly basis to describe senescence. The leaf longevity factor, δ, of the GDMM (Table 1) was then adjusted using the previously measured values of {*i*_*top*_, ϕ_*GDD*_, *MA*_*big*_} and the senescence fraction. Finally, *GLAI*_*field*_ of a microplot was obtained by simulating the GLAI with the GDMM for the corresponding GDD and the parameters {*d, i*_*top*_, ϕ_*GDD*_, *MA*_*big*_, δ} of the microplot.

### Multispectral Image Acquisition From the UAV and Data Processing

A hexacopter UAV was used for nine (2016) and eleven (2017) flights on dates selected to represent the dynamics of GLAI over the growth cycle. Furthermore, the UAV was flying always under clear sky and low to medium wind speed conditions. The AIRPHEN multispectral camera (www.hiphen-plant.com) was fixed on a two-axis gimbal to point downward, vertically. The device is composed of six cameras equipped with an 8 mm focal length lens. They record 1,280 × 960 pixel images with 10 nm spectral resolution bands centered at 450, 532, 568, 675, 730, and 850 nm. The integration time of each camera was adjusted automatically to minimize saturation and maximize the range of variation. Images were acquired continuously during the flight at a 1 Hz frequency. The flight plan was designed to ensure 80% overlap both across and along the track. The flight altitude was fixed to 60 m to provide a ground spatial resolution of around 2.5 cm.

Radiometric calibration was performed using a 3 m^2^ reference panel. In addition, nine circular panels of 60 cm diameter were placed within the field and used as ground control points (GCPs). The positions of the GCPs were measured with RTK-GPS, providing an accuracy of around 2 cm.

The multispectral images were first corrected for the vignetting effect, and then co-registered using the method proposed by Rabatel and Labbé (2016). Agisoft Photoscan software (v1.2.2, 2015, Agisoft LLC., Russia) was used to find the position and orientation of the camera for each individual image. This information was then used to project the images onto the ground and to extract the microplots based on the coordinates of their corners measured previously. The GCPs were used to ensure good geometric consistency between the projected images and the microplots coordinates. Finally, radiometric calibration was applied to compute the bidirectional reflectance factor, *r*_*b*_, in the six bands *b* using the recorded integration time and the images captured over the radiometric panel. To limit the impact of possible variations of illumination conditions during the flight, normalized reflectances, $r_{b}^{*}$ were computed by dividing the reflectance in each band by that observed in the near infrared: $r_{b}^{*}=\frac{r_{b}}{r_{850}}$ with *b* ∈ {450, 532, 568, 675, 730}. More details can be found in Jay et al. (2018).

### Estimation of GLAI of Each Microplot (GLAI_TF_) Using Transfer Functions Calibrated Over the Reference Microplots

For each flight, an empirical transfer function was calibrated between *GLAI*_*field*_ and the corresponding values of $\left\{r_{450}^{*},\;r_{532}^{*},\;r_{568}^{*},\;r_{675}^{*},\;r_{730}^{*},\;d,\;i_{top},\;\phi_{GDD}\right\}$ used as predictors. To improve the performance of the transfer function, variables {*d, i*_*top*_, ϕ_*GDD*_} were added to the normalized reflectances $\left\{r_{450}^{*},\;r_{532}^{*},\;r_{568}^{*},\;r_{675}^{*},\;r_{730}^{*}\right\}$ since they were available for all the microplots and expected to impact the GLAI (Supplementary Figure 2). Ridge regression (Hoerl and Kennard, 1970) was used to calibrate the transfer functions (**Equation 4**) to deal with possible multicollinearity between predictors.

(4) $$\begin{array}{l} GLAI_{field}=\mu +\overset{5}{\underset{j=1}{\sum }}\alpha_{j}r_{b_{j}}^{*}+\alpha_{6}d+\alpha_{7}i_{top}+\alpha_{8}\phi_{GDD}+E \end{array}$$

where μ is the intercept, α_*j, j*∈[[1, 5]]_ the effect of normalized reflectance $r_{b_{j}}^{*}$ observed in the wavelength *b*_*j*_ (*b* = {450, 532, 568, 675, 730}), α_6_ the effect of the density *d*, α_7_ the effect of the final number of leaf *i*_*top*_ and α_8_ the effect of the phyllochron ϕ_*GDD*_. *E* is the random residual, with $E\sim N\left(0,\sigma^{2}I\right)$ and *I* the identity matrix. Ridge regressions were computed with the *glmnet R package v2.0-13* (Friedman et al., 2010; R Core Team, 2017).

To evaluate the relevance of the additional variables {*d, i*_*top*_, ϕ_*GDD*_} as predictors jointly with the normalized reflectance $\left\{r_{450}^{*},\;r_{532}^{*},\;r_{568}^{*},\;r_{675}^{*},\;r_{730}^{*}\right\}$, transfer functions using only the multispectral data, TF_UAV_, or only the additional variables, TF_prior_, were considered in addition to the transfer function TF_full_ using all eight variables. A leave-one-out cross-validation approach (Efron and Tibshirani, 1993) was used to evaluate the prediction performance of the three different transfer functions by computing the Root Mean Squared Error of Prediction (RMSEP) and the Relative RMSEP (RRMSEP).

The calibration domain, defined as the distribution of the predictors $\left\{r_{450}^{*},\;r_{532}^{*},\;r_{568}^{*},\;r_{675}^{*},\;r_{730}^{*},\;d,\;i_{top},\;\phi_{GDD}\right\}$ over the reference microplots, was compared to the application domain, defined as the distribution of the predictors over the whole experiment. The convex hull of the calibration domain was first computed over the reference microplots using the *R package geometry v0.3-6* (Habel et al., 2015). It was then expanded by 5% over all eight dimensions, assuming that in the vicinity of the calibration domain, the transfer function should behave with similar performance. For each date, the percentage of microplots included in the calibration and extended calibration domain was computed to evaluate the representativeness of the reference microplots used to calibrate the transfer function.

Once calibrated over the reference microplots for a given flight date, the full transfer function TF_full_ was finally applied to the whole experiment to predict the GLAI value, *GLAI*_*TF*_, for each microplot on the date considered.

### Estimation of MA_big_ and *δ* Over the Whole Experiment

For each microplot, the unknown parameters of the GDMM, {*MA*_*big*_, δ} were estimated by inverting the GDMM, i.e., by fitting the GDMM over the *GLAI*_*TF*_ values for the nine (2016) or eleven (2017) flight dates. The other three parameters were either measured directly (Table 1) over each microplot (*d*) or over the 2016 WW treatment ({*i*_*top*_, ϕ_*GDD*_}), since they were considered to be dependent only on the genotype. A Look-Up-Table approach was used since it is easy to implement, runs fast and avoids trapping in local minima. It consisted in simulating the GLAI value with the GDMM for 10,000 combinations of the two parameters to be estimated, {*MA*_*big*_, δ}, for each flight date and each set of {*d, i*_*top*_, ϕ_*GDD*_}. Parameters {*MA*_*big*_, δ} were randomly drawn using uniform distributions within their possible range of variation: $2.10^{-2}\;{\text{m}}^{2}<MA_{big}<8.10^{-2}\;{\text{m}}^{2}$; 250°C.d < δ < 2, 000°C.d. These ranges were estimated based on the reference plot variations. A cost function, *J*, computed for the 10 000 simulations of the GDMM, quantified the agreement between the simulated GLAI, *GLAI*_*sim*_, and the GLAI estimated with the transfer function, *GLAI*_*TF*_:

(5) $$\begin{array}{l} J=\frac{1}{n}\overset{n}{\underset{t=1}{\sum }}{\left(\frac{GLAI_{TF}\left(t\right)-GLAI_{sim}\left(t\right)}{\sigma \left(t\right)}\right)}^{2} \end{array}$$

where σ(*t*) is the corresponding uncertainty of both *GLAI*_*TF*_ estimation and GDMM for the date *t*, approximated as:

(6) $$\begin{array}{l} \sigma \left(t\right)=\{\begin{array}{cc} 0.1 & if\;GLAI_{TF}\left(t\right)<1\\ 0.1\times GLAI_{TF}\left(t\right) & if\;GLAI_{TF}\left(t\right)\geq 1 \end{array} \end{array}$$

The solution was computed as the average of the combinations leading to *J* ≤ 1, i.e., for which the difference between the simulated *GLAI*_*sim*_ and *GLAI*_*TF*_ is smaller than the associated uncertainty σ (Diner et al., 1999; Zhang et al., 2000; Wang et al., 2001).

The estimated values of {*MA*_*big*_, δ} were used along with the known GDMM parameters {*d, i*_*top*_, ϕ_*GDD*_} to estimate the GLAI values, *GLAI*_*est*_, continuously from emergence to harvest. The Area Under the Curve (*AUC*) of *GLAI*_*est*_ was also computed from emergence to harvest to account for both the magnitude and duration of GLAI dynamics.

### Statistical Analysis

#### Adjusted Means and Broad-Sense Heritability

Best linear unbiased estimates of the genotypes (adjusted means) were estimated from a linear mixed model fitted for each combination of trait, year, and treatment.

(7) $$\begin{array}{l} Y_{ij}=\mu +b_{i}+g_{j}+E_{ij} \end{array}$$

Where *Y*_*ij*_ is the phenotypic value, μ the overall mean, *b*_*i*_ the fixed effect of replicate *i* and *g*_*j*_ the fixed effect of genotype *j*. *E* is the random residual, $E\sim N\left[0,\sigma^{2}\left(R_{r}⊗R_{c}\right)\right]$ with *R*_*r*_ and *R*_*c*_ the correlation matrices for the row and column first order autoregressive processes, respectively, as proposed by Gilmour et al. (1997).

Broad-sense heritability (*H*^2^) was computed following Cullis et al. (2006):

(8) $$\begin{array}{l} H^{2}=1-\frac{{\overset{-}{\nu }}_{\Delta BLUP}}{2\sigma_{G}^{2}} \end{array}$$

where $\sigma_{G}^{2}$ is the genetic variance and ${\overset{-}{\nu }}_{\Delta BLUP}$ the mean variance of a difference between two BLUPs (Best Linear Unbiased Predictions). **Model 7** was fitted again, considering the genotype as a random effect, to estimate $\sigma_{G}^{2}$ and ${\overset{-}{\nu }}_{\Delta BLUP}$. All linear models were fitted using the *R package ASReml-R v3.0* (Butler et al., 2009).

#### Impact of GLAI on Grain Yield and Drought Stress Tolerance

A linear model (**9**) was used to evaluate the effect of the estimated GLAI traits *MA*_*big*_, δ and *AUC* on grain yield in each environment. The effect of genotype earliness was also considered in the model.

(9) $$\begin{array}{l} Y_{i}=\mu +af_{i}+bAUC_{i}+cMA_{big,i}+d\delta_{i}+E_{i} \end{array}$$

where *Y*_*i*_ is the grain yield or its components of genotype *i*, μ is the intercept, *a* the effect of the flowering date *f*, *b* the effect of *AUC*, *c* the effect of *MA*_*big*_ and *d* the effect of the δ. *E* is the random residual, with $E\sim N\left(0,\sigma^{2}I\right)$ and *I* the identity matrix.

The drought response of a trait (GLAI dynamics or grain yield and its components) was defined as the normalized difference between the value of this trait in the WW condition and in the WD condition:

(10) $$\begin{array}{l} ND_{Y}=\frac{Y_{WW}-Y_{WD}}{Y_{WW}} \end{array}$$

where *Y*_*WW*_ and *Y*_*WD*_ are respectively the trait values in the WW and WD treatments of the same year. For *GLAI*_*est*_, the normalized difference was computed every 25°C.d from 50 to 2,000°C.d for each year and a hierarchical clustering was performed using Ward's distance and the *stats R package*. The inertia gain method was used to choose the number of clusters. This allowed identifying groups of genotypes that exhibit similar changes in the shape of GLAI dynamics between WW and WD conditions.

The effect of dynamics' drought response patterns on grain yield stability was finally tested jointly with the genotype precocity effect in the following model:

(11) $$\begin{array}{l} Y_{ij}=\mu +af_{ij}+C_{j}+E_{ij} \end{array}$$

where *Y*_*ij*_ is the normalized difference of grain yield (or its components) for the individual *i* in the cluster *j*, μ the intercept, *a* the effect of the precocity *f* and *C*_*j*_ the effect of cluster *j*. *E* is the random residual, with $E\sim N\left(0,\sigma^{2}I\right)$ and *I* the identity matrix.

## Results and Discussion

### Environmental Conditions and Field Measurements

**Figure 1:**
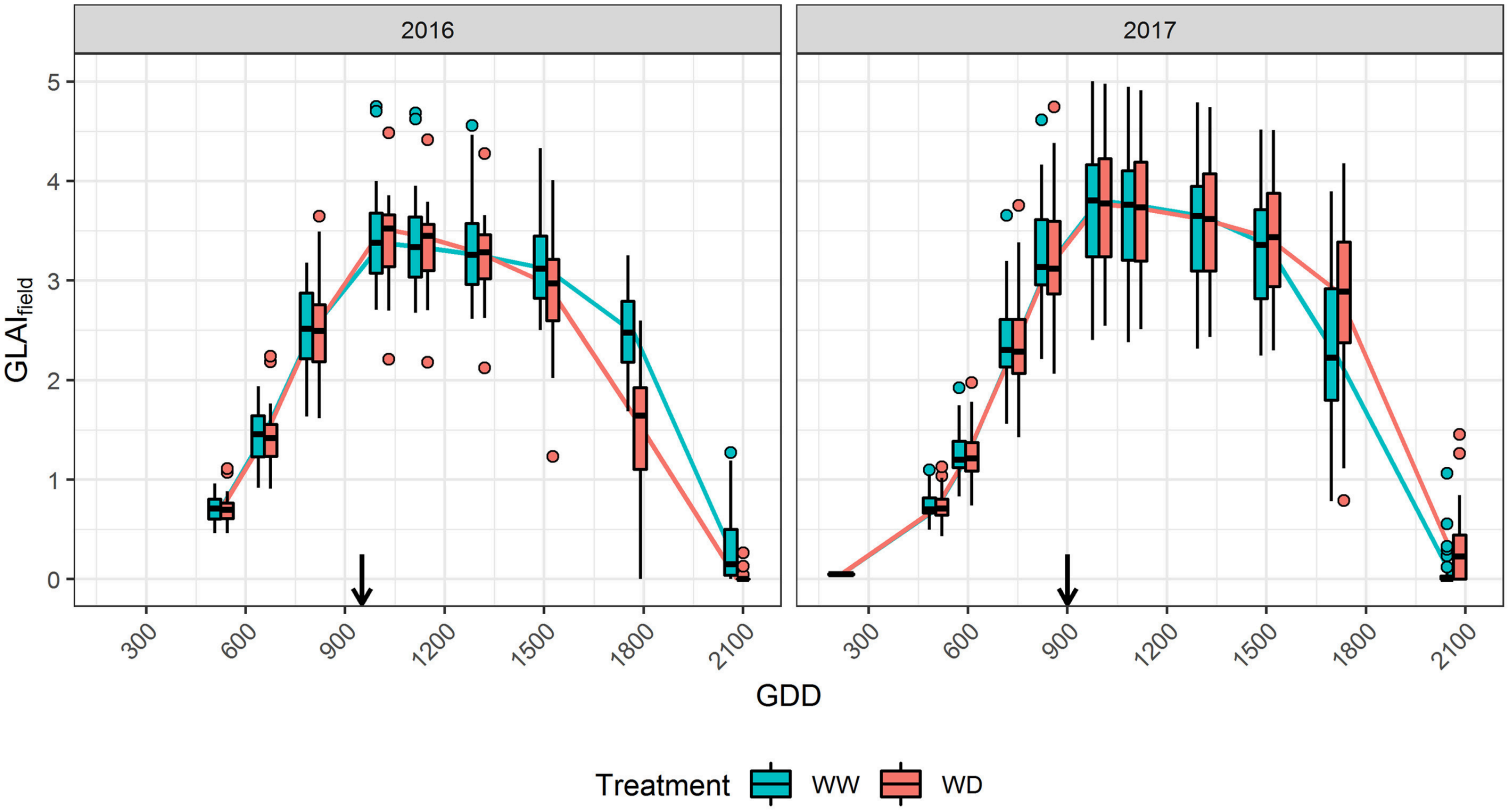
Effect of drought stress on *GLAI*_*field*_ dynamics in 2016 and 2017. Boxplots are built from the reference microplots data for each flight date. The horizontal line in the boxplots corresponds to the median, while the diamond corresponds to the mean. The lower and upper hinges show the first and third quartiles, and the whiskers correspond to 1.5 times the inter-quartile range or to the most extreme value, whichever is smallest. Dots represents values outside this range. Black arrows indicate flowering time.

In 2016, soil moisture monitoring and water balance showed that the flowering drought stress was limited for the WD treatment due to rainfalls just around flowering, and that only a light stress occurred from 8^th^ July (≈800°C.d) to 5^th^ August (≈1,250°C.d). Nevertheless, a more severe stress occurred during the grain filling stage from approximately 5^th^ August to 25^th^ August (≈1,550°C.d). The water stress impacted the reference microplots GLAI with an earlier senescence (Figure 1). It also resulted in a 40% loss for grain yield over the whole panel in WD compared to the WW treatment (Table 2), explained by a 20% reduction of both the kernel number and the thousand kernel weight. In 2017, water stress took place in the WD treatment around flowering, from 8^th^ July (≈750°C.d) to 29^th^ July (≈1,100°C.d), delayed the senescence (Figure 1) and reduced yield by 21% mostly because of a reduction of the kernel number. For both years, no water stress was detected in the WW condition. However, in 2017 soil crusting impacted stand establishment in both conditions, with an actual density of around 8 plants.m^−2^ in WW and 7.5 plants.m^−2^ in the WD treatment. Comparison of GLAI dynamics between years shows that maximum GLAI was significantly higher and more variable in 2017 than in 2016.

**Table 2:** Climatic conditions, yield and its components in 2016 and 2017 for the Well-Watered (WW) and Water-Deficient (WD) conditions.

	**2016**		**2017**	
	**WW**	**WD**	**WW**	**WD**
Cumulated global radiation (MJ.m^−^^2^)	2,969		2,990	
Average day temperature (°C)	22.0		22.8	
Average night temperature (°C)	16.3		16.9	
Vapor pressure deficit (kPa)	1.08		1.28	
Rainfall (mm)	358		170	
Irrigation (mm)	310	109	365	235
Yield at 15% moisture (q.ha^−1^)	95	59	94	75
Kernel number per square meter	3,522	2,804	3,257	2,597
Thousand kernel weight at 15% moisture (g)	269	212	289	288
Harvest grain moisture (%)	25	23	23	23

### Combining UAV Observations and GDMM Enables Accurate and High-Throughput Phenotyping of GLAI Dynamics and Underlying Traits

The phenotyping method developed in this study is divided in two steps. First, transfer functions are calibrated using UAV observations and field reference measurements, and are then used to predict GLAI of the whole panel for each flight date (*GLAI*_*TF*_). Secondly, the GDMM is inverted based on *GLAI*_*TF*_ data to finally provide the continuous GLAI dynamics (*GLAI*_*est*_) and two underlying traits (*MA*_*big*_ and δ).

**Figure 2:**
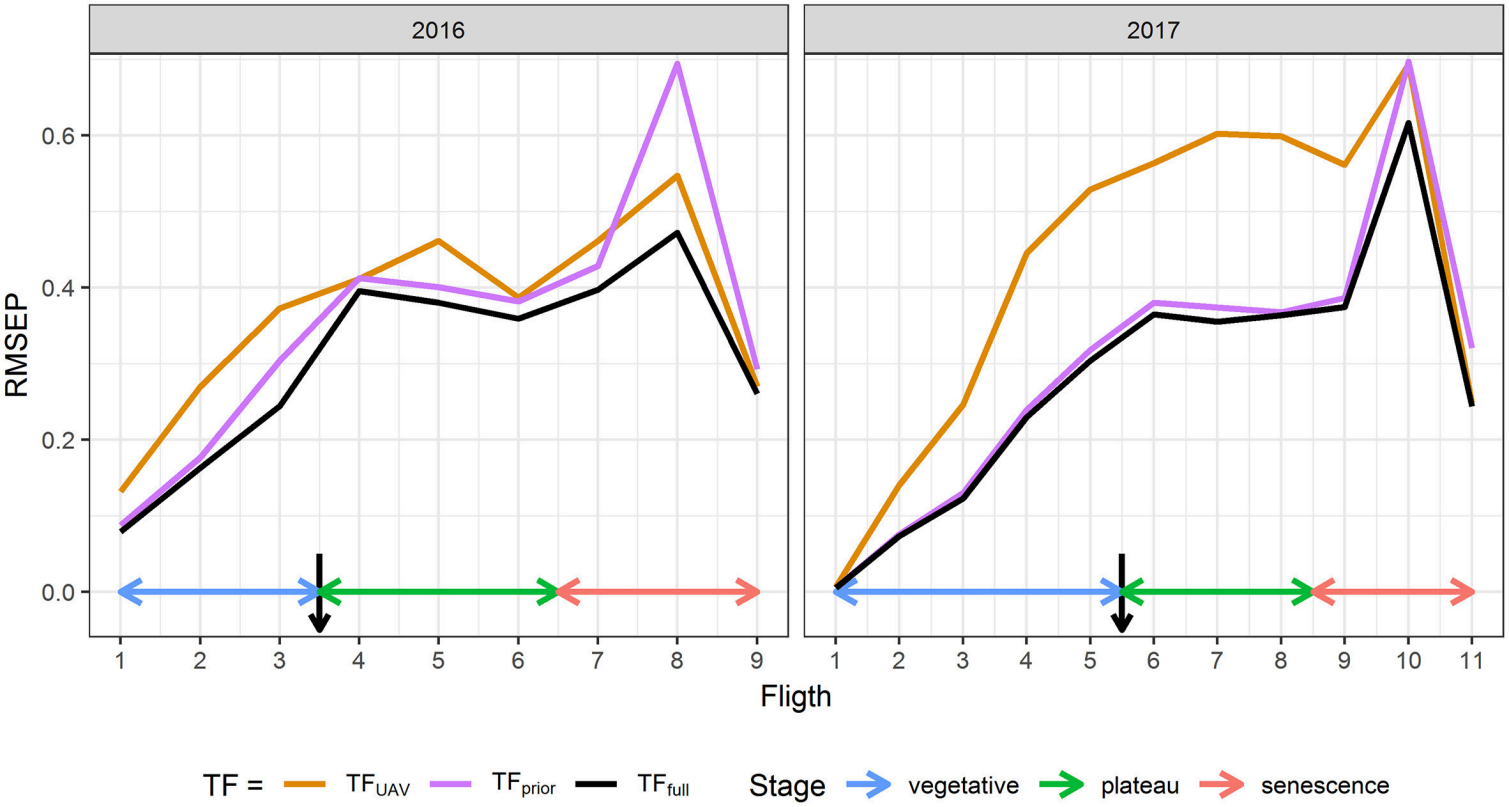
Comparison between the Root Mean Squared Error of Prediction (RMSEP) of TF_full_, TF_UAV_, and TF_prior_ for each flight date in 2016 and 2017. TF_full_ is the transfer function based on the eight predictors $\left\{r_{450}^{*},\;r_{532}^{*},\;r_{568}^{*},\;r_{675}^{*},\;r_{730}^{*},d,\;i_{top},\;\phi_{GDD}\right\}$, while TF_UAV_ is based only on $\left\{r_{450}^{*},\;r_{532}^{*},\;r_{568}^{*},\;r_{675}^{*},\;r_{730}^{*}\right\}$ and TF_prior_ only on {*d, i*_*top*_, ϕ_*GDD*_}. Black arrows indicate flowering time.

*GLAI*_*TF*_ estimated from TF_full_ using the eight predictors $\left\{r_{450}^{*},\;r_{532}^{*},\;r_{568}^{*},\;r_{675}^{*},\;r_{730}^{*},d,i_{top},\phi_{GDD}\right\}$ agrees closely with the reference *GLAI*_*field*_ over the cycle for both years (Supplementary Figure 3) with a high coefficient of determination (R^2^≈0.95), and a low Root Mean Squared Error (RMSE≈0.3, Relative RMSE≈13%). Moreover, the RMSEP shows that the prediction error is low and similar to the RMSE of the reference sample with 0.01 ≤ RMSEP ≤ 0.62 for 0 ≤ GLAI ≤ 5 depending on the flight date (Figure 2). The RRMSEP was close to 10% for both years throughout the whole growth cycle, except for the two last dates, because RMSEP remained fairly constant but *GLAI*_*field*_ decreased due to senescence (Figure 1). This increase in RRMSEP for the last dates may be due to confounding effects originating from the similarity of senescent vegetation and bare soil spectra (Girard and Girard, 2003).

The transfer functions in **Equation 4** combine two types of GLAI predictors. We further evaluated the contribution of these two types of predictors by considering two other transfer functions: TF_UAV_ when using only the five normalized reflectance $\left\{r_{450}^{*},\;r_{532}^{*},\;r_{568}^{*},\;r_{675}^{*},\;r_{730}^{*}\right\}$ and TF_prior_ when using only the three additional predictors {*d, i*_*top*_, ϕ_*GDD*_}. Results clearly show that the transfer functions using the eight predictors, TF_full_, perform better than the two other transfer functions (Figure 2). This is particularly clear for the early stages, when {*d, i*_*top*_, ϕ_*GDD*_} are the key drivers of the GLAI dynamics (Supplementary Figure 2). Furthermore, during the end of the vegetative and plateau periods, when GLAI was high, saturation of the reflectance signal could degrade GLAI retrieval from the multispectral data (Baret and Guyot, 1991). This explains the higher RMSEP of TF_UAV_ in 2017, especially during the plateau period, because the GLAI was higher in 2017 as compared to 2016 (Figure 1). Nevertheless, during the senescence period the impact of parameter δ is dominant (Supplementary Figure 2) and UAV observations bring valuable information that significantly improve GLAI predictions. Indeed, using the spectral predictors leads to moderate RRMSEP values during the critical period of the senescence because it limits RMSEP inflation when the GLAI values decrease substantially (Figures 1, 2).

**Figure 3:**
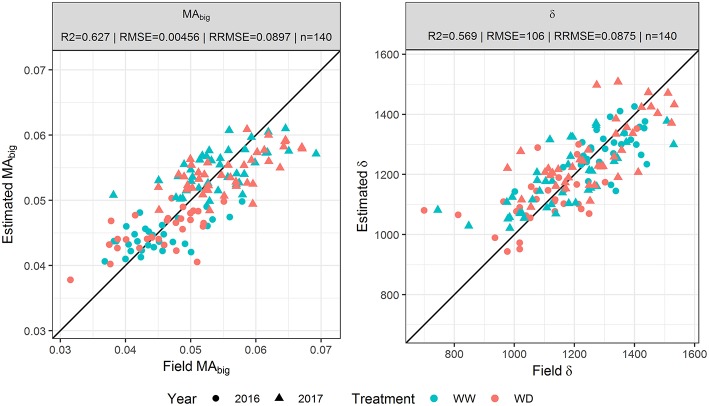
Correlation between the biggest leaf area (*MA*_*big*_) and leaf longevity (δ) assessed in the field and estimated by inverting the GLAI Dynamics Maize Model based on GLAI dynamics obtained from TF_full_. TF_full_ is the transfer function based on the eight predictors $\left\{r_{450}^{*},\;r_{532}^{*},\;r_{568}^{*},\;r_{675}^{*},\;r_{730}^{*},d,\;i_{top},\;\phi_{GDD}\right\}$.

Parameters {*MA*_*big*_, δ} of the GDMM were adjusted over the *GLAI*_*TF*_ dynamics estimated from each of the three transfer functions. The GDMM was then run with the estimated values of {*MA*_*big*_, δ} to get *GLAI*_*est*_ that describes the continuous GLAI dynamics of each microplot. Results show that *GLAI*_*est*_ is highly correlated to *GLAI*_*TF*_ for the three transfer functions with R^2^ ≥ 0.98 and RRMSE ≤ 0.08. However, the estimation accuracy of parameters {*MA*_*big*_, δ} varies greatly depending on the use of *GLAI*_*TF*_ dynamics from TF_full_, TF_UAV_ or TF_prior_ to invert the GDMM. Indeed, when using TF_full_ estimates to fit the GDMM, the parameters {*MA*_*big*_, δ} are retrieved with a good accuracy (*R*^2^≈0.6 and RRMSE < 9%, Figure 3), although the R^2^ values are relatively low for *MA*_*big*_ when computed for each individual year (Table 3) due to the restricted range of variation observed in 2016 and 2017. Conversely, inverting the GDMM based on *GLAI*_*TF*_ dynamics obtained from TF_UAV_ or TF_prior_ significantly degraded the accuracy of {*MA*_*big*_, δ} retrieval (Table 3). Differences of retrieval accuracy between *GLAI*_*est*_ dynamics and {*MA*_*big*_, δ} when using TF_UAV_ and TF_prior_ estimates to invert the GDMM are not surprising, as numerous combinations of parameters can lead to the same expected dynamics.

**Table 3:** Coefficient of determination (R^2^), Root Mean Square Error (RMSE) and Relative RMSE (RRMSE) values obtained when using three sets of predictors in the transfer functions to estimate the biggest leaf area (*MA*_*big*_) and leaf longevity (δ) by inverting the GLAI Dynamics Maize Model.

**Name**	**MA**_*******big*******_						**δ**					
	**2016**			**2017**			**2016**			**2017**		
	**R^**2**^**	**RMSE**	**RRMSE (%)**	**R^**2**^**	**RMSE**	**RRMSE (%)**	**R^**2**^**	**RMSE**	**RRMSE (%)**	**R^**2**^**	**RMSE**	**RRMSE (%)**
TF_prior_	0.13	0.0050	11	0.23	0.0052	10	−0.04	163	14	0.24	141	12
TF_UAV_	0.18	0.0047	10	−0.81	0.0077	14	0.40	126	11	0.62	93	8
TF_full_	0.36	0.0043	10	0.35	0.0048	9	0.58	104	9	0.56	107	9

TF_full_ is the transfer function based on the eight predictor $\left\{r_{450}^{*},\;r_{532}^{*},\;r_{568}^{*},\;r_{675}^{*},\;r_{730}^{*},d,\;i_{top},\;\phi_{GDD}\right\}$, while TF_UAV_ is based only on $\left\{r_{450}^{*},\;r_{532}^{*},\;r_{568}^{*},\;r_{675}^{*},\;r_{730}^{*}\right\}$ and TF_prior_ only on {d, i_top_, ϕ_GDD_}.

Currently, UAV is the only phenotyping tool able to deliver both high spatial resolution (Tattaris et al., 2016) and throughput (Madec et al., 2017; Yang et al., 2017a) in the field. These characteristics, added to its reasonable cost (Reynolds et al., 2018), explain its growing popularity in the last decade (Yang et al., 2017a). Although numerous studies have been conducted about crop phenotyping from UAV, most of them focus on vegetation indices to describe the evolution of the canopy. Very few studies such as Lelong et al. (2008), Potgieter et al. (2017) and Yao et al. (2017), aimed at characterizing the GLAI dynamics throughout the whole cycle from UAV imagery. Moreover, these studies dealt with few genotypes and/or microplots and delivered GLAI estimates on a limited number of time points distributed throughout the cycle. In this study, we proposed an innovative way of developing transfer functions, consisting in using spectral predictors $\left\{r_{450}^{*},\;r_{532}^{*},\;r_{568}^{*},\;r_{675}^{*},\;r_{730}^{*}\right\}$ concurrently with additional known variables {*i*_*top*_, ϕ_*GDD*_, *d*} to predict GLAI dynamics of a large panel. The resulting transfer functions provided good accuracy compared to other studies where GLAI was retrieved from remote sensing observations (Haboudane et al., 2004; Walthall et al., 2004; Verger et al., 2014; Verrelst et al., 2015; Kang et al., 2016), especially given the small size of the microplots, and the diversity of the genotypes characterized. However, possible residual genotypic effects related to differences in leaf orientation or aggregation may be present because a unique transfer function was used for all genotypes on each date. Additional view directions or the use of a proper 3D model of canopy architecture may help to solve this structure effect problem and further improve the prediction accuracy (López-Lozano et al., 2007; Baret et al., 2010; Liu et al., 2016).

Inversion of the GDMM also appears to be a valuable approach as it provides continuous dynamics that are more biologically meaningful than time point estimates or dynamics obtained by adjusting purely mathematical functions (Koetz et al., 2005). Two fine scale traits: the area of the biggest leaf (*MA*_*big*_) and leaf longevity parameters (δ) were also retrieved with a satisfying accuracy, providing that priori information was available. However, the temporal sampling of *GLAI*_*TF*_ might have a strong impact on the retrieval performances of the inversion process (Kalogiros et al., 2016). The flight dates were approximately evenly distributed along the growth cycle in our study, but a simulation approach using the GDMM model could be used to determine the optimal temporal sampling.

The good performance of our approach is partly explained by the good representativeness of the reference plots as compared to the whole experiment. The convex hull of the calibration domain computed for the eight predictors $\left\{r_{450}^{*},\;r_{532}^{*},\;r_{568}^{*},\;r_{675}^{*},\;r_{730}^{*},\;d,\;i_{top},\;\phi_{GDD}\right\}$ and extended by 5% to account for the associated uncertainties included 87% (2016) and 91% (2017) of the whole dataset. These results show that ground measurement for only 5% of the total number of microplots is sufficient to accurately predict the GLAI dynamics and its two underlying traits on the whole experiment. However, in our case the genotypes were selected to represent a wide range of variation based on prior knowledge gained on the genotype characteristics, which may not be possible in all the situations.

This method reduced the phenotyping time by a factor of about 20 compared to fully ground-based phenotyping. This makes possible for only one person to perform all the ground measurements and assess GLAI dynamics of large populations that were previously unmanageable. To our best knowledge, this study is one of the first describing a field phenotyping method to characterize the maize GLAI dynamics continuously throughout the cycle, with sufficient throughput and accuracy to fit breeding and genetic studies requirements.

### Unraveling GLAI Dynamics Response to Contrasted Environmental Conditions

**Figure 4:**
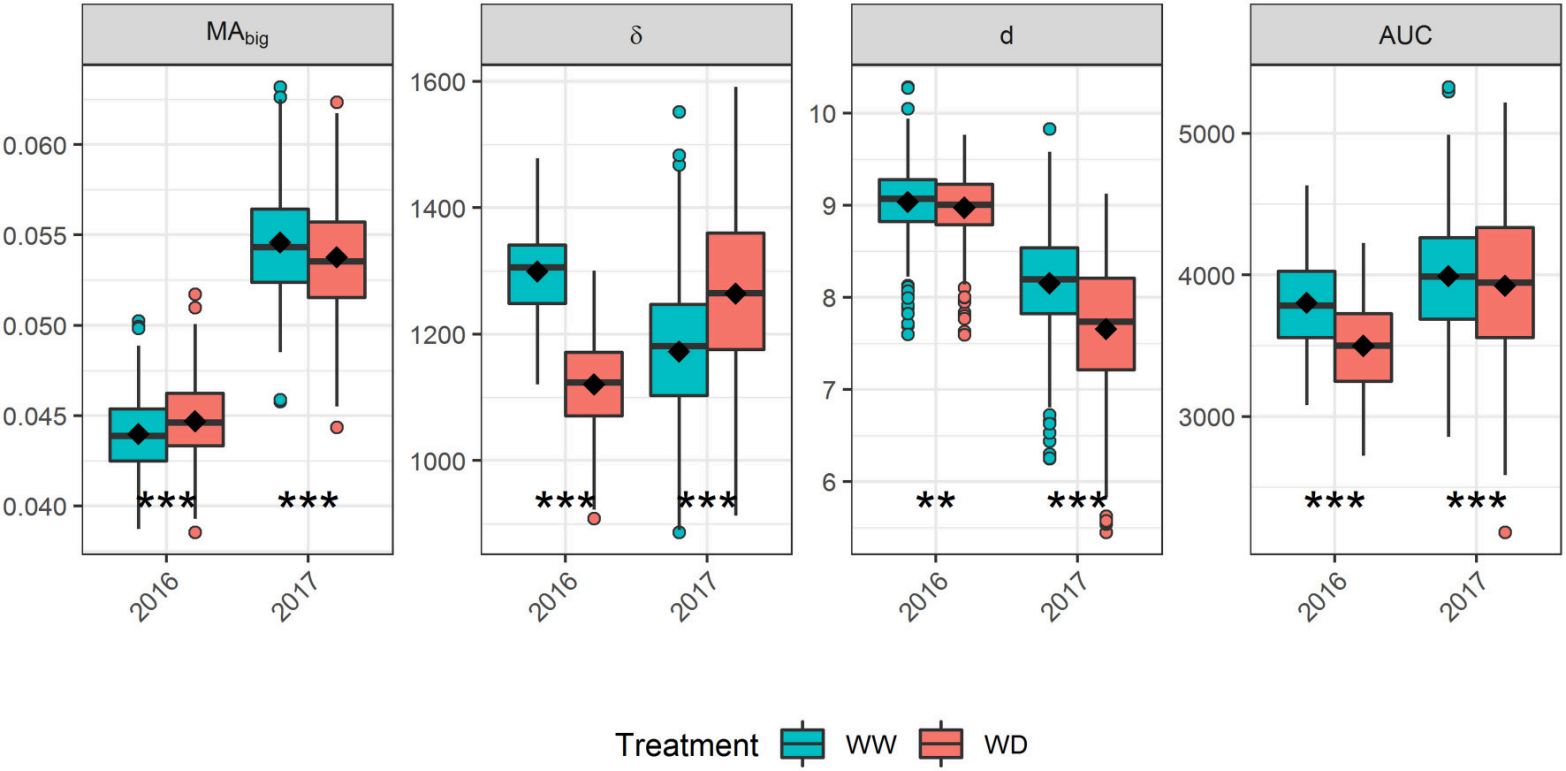
Impact of drought stress on the biggest leaf area (*MA*_*big*_), leaf longevity (δ), density (*d*) and the Area Under the Curve (*AUC*) in 2016 and 2017. Boxplots are built from adjusted means. The horizontal line in the boxplots corresponds to the median, while the diamond corresponds to the mean. The lower and upper hinges show the first and third quartiles, and the whiskers correspond to 1.5 times the inter-quartile range or to the most extreme value, whichever is smallest. Dots represent values outside this range. Asterisks indicate a significant difference of means between treatments based on a paired *t*-test: ^***^ *p* ≤ 0.001; ^**^ *p* ≤ 0.01.

The differences observed for the GLAI dynamics between water treatments (Figure 1) can be better understood by analyzing its underlying components. In 2016, *AUC* was greatly reduced under the WD condition compared to the WW condition (-8%, Figure 4) which is mainly explained by the decrease of δ (Supplementary Figure 4). This decreased stay-green under drought (-13.7%) is consistent with the timing of the water stress as reported by previous results (Kamara et al., 2003; Çakir, 2004; Young et al., 2004; Li et al., 2018; Mangani et al., 2018). On the contrary, in 2017 the reduced *MA*_*big*_ and density, *d* was compensated by an increase of δ, leading to almost similar *AUC* values for both treatments (−1.7%). Conversely to 2016, this increased stay-green (+7.9%) under drought is surprising. A possible explanation is that the reduced *MA*_*big*_ and *d* probably led to an increased light homogeneity in the canopy. Indeed, better light distribution in the canopy strata have been shown to delay the canopy senescence (Borrás et al., 2003; Huang et al., 2017; Yang et al., 2019). The difference in plant density between treatments was negligible in 2016 compared to 2017, supporting this explanation.

In both years, δ seems more affected by drought stress than *MA*_*big*_ (Figure 4) because the water stress started at the end of leaf growth, allowing an almost optimal setup of the plant leaf area (Çakir, 2004; Li et al., 2018). Thus, δ seems to be a promising trait for exploring the impact of flowering or grain-filling drought stress on GLAI dynamics. However, the small variation of *MA*_*big*_ between treatments should be opposed to the strong increase that occurred in 2017 as compared to 2016 (Figure 4). This increase might be explained by the lower density observed in 2017 (Supplementary Figure 4), with less competition for resources resulting in bigger leaves. Therefore, *MA*_*big*_ could be a valuable trait for studying impact of other stress on GLAI dynamics, including early drought and high plant density.

**Figure 5:**
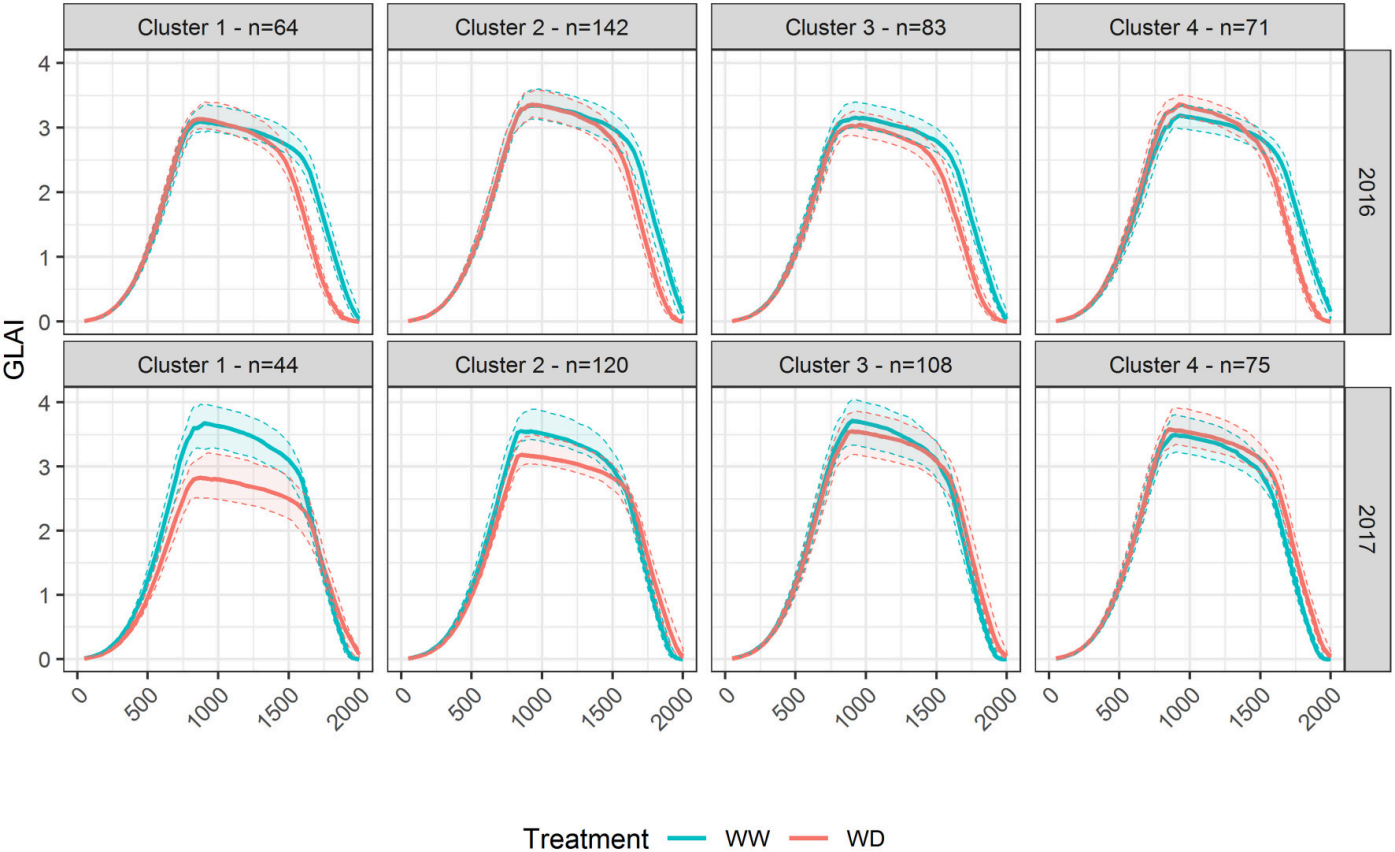
GLAI dynamics response to drought representative of the panel diversity in 2016 and 2017. The hierarchical clustering applied to the normalized difference of GLAI dynamics in Well-Watered and Water-Deficient treatments resulted in four distinct clusters in both years. The thick line represents the median of *GLAI*_*est*_ for the genotypes in the corresponding cluster, while the thin dashed lines show the first and third quartiles. N is the number of genotypes in each cluster. Data are adjusted means.

GLAI response to water stress was further analyzed by applying a hierarchical clustering to the genotypic relative differences of GLAI dynamics between WW and WD treatments. It revealed four different patterns of response to drought stress in both years, representing between 13% (Cluster 1 in 2017) and 39% (Cluster 2 in 2016) of the panel (Figure 5). In 2016, all patterns showed earlier senescence under the WD condition. The differences are on the timing of the senescence onset as well as the impact of drought on the maximum GLAI value. Surprisingly, the fourth cluster showed a larger amplitude of GLAI dynamics under the WD condition. In 2017, the maximum GLAI value was impacted by drought stress with a variable magnitude amongst the four clusters (Figure 5), while senescence was only slightly delayed.

The patterns of GLAI dynamics' drought response is significantly linked to the stability of grain yield (Supplementary Figure 5). Interestingly, the effect of GLAI dynamics response was not due to a drought escape allowed by precocity which was considered in the linear model. In 2016, the dynamics clusters explained 5.7, 5.2, 7.2, and 10% of the drought tolerance of grain yield, kernel number, thousand kernel weight and harvest grain moisture, respectively. A higher amplitude of dynamics under the WD condition (Figure 5, cluster 4) was associated with better stability of grain yield and kernel number under drought stress (Supplementary Figure 5). Also, a maximum stay-green led to a limited thousand kernel weight loss and a higher harvest grain moisture (clusters 2, 3, and 4). These results are consistent with the timing of the drought: the flowering stress impacted the maximum GLAI value and the establishment of the kernel number, while the grain filling stress affected the thousand kernel weight and harvest grain moisture through earlier senescence (Çakir, 2004; Li et al., 2018; Mangani et al., 2018).

In 2017, the clusters explained a smaller part of the grain yield (4%) and kernel number (6.4%) stability while the cluster effect was not significant for the thousand kernel weight and harvest grain moisture. Cluster 4 exhibited very similar GLAI dynamics under both treatments, resulting in the smallest impact of the drought on grain yield and kernel number (Supplementary Figure 5). For the three other clusters, the magnitude of the reduction of the maximum GLAI value led to a corresponding decrease of grain yield and kernel number. Although these results seem in good agreement with the timing of the stress experienced in 2017, it is not possible to clearly attribute either the grain yield variation or the response of the GLAI dynamics to the flowering drought stress rather than to the reduced plant density.

Here, *MA*_*big*_, δ and *AUC* were used to decipher an average response of GLAI dynamics to drought stress, while clustering was used to identify groups of genotypes with typical pattern of drought response. Both approaches bring valuable insights to understand the GLAI dynamics adaptation to environmental conditions. Despite the relatively simple clustering method used, our study demonstrated that the timing and magnitude of GLAI values were consistent with the timing and magnitude of the water stress experienced with possible consequences on the thousand kernel weight (2016) and kernel number (2016 and 2017) (Supplementary Figure 5). Such clustering approach was applied to temporal series of observations over maize (Han et al., 2018; Su et al., 2019) and rice (Campbell et al., 2015). However, it is not yet widely used because of the still limited number of studies based on high temporal phenotyping of many genotypes.

A better understanding of drought stress impact on GLAI dynamics and its underlying traits is a first step toward the design of new ideotypes for maize breeding. Comprehensive crop models like APSIM, DSSAT, or STICS, have been used to study how specific traits influence grain yield of a reference genotype under a large range of current and future environments, and predict the best combination of traits to maximize productivity (Hammer et al., 2005; Chenu et al., 2008; Harrison et al., 2014; Parent et al., 2018). However, these models are sometimes inaccurate, especially when dealing with stressing environments (Rötter et al., 2015). Leaf Area Index assessed locally have been used to constrain or update crop model predictions for specific experiments and showed good results (Casa et al., 2012; Jégo et al., 2012). The rapid accumulation of field GLAI data provided by high-throughput phenotyping, coupled with proper envirotyping, will allow to accurately link average GLAI response to environmental factors and improve crop models' calibration. This in turn will contribute to the design of more valuable ideotypes. While high-throughput phenotyping platform under controlled conditions are efficient to estimate leaf related traits (Cabrera-Bosquet et al., 2016) and have already been used to calibrate crop models (Parent et al., 2018), field phenotyping would allow to explore a wider but also more realistic range of environmental conditions (White et al., 2012).

### GLAI Traits Are Promising Traits for Maize Breeding Under Optimal and Water Limited Conditions

**Figure 6:**
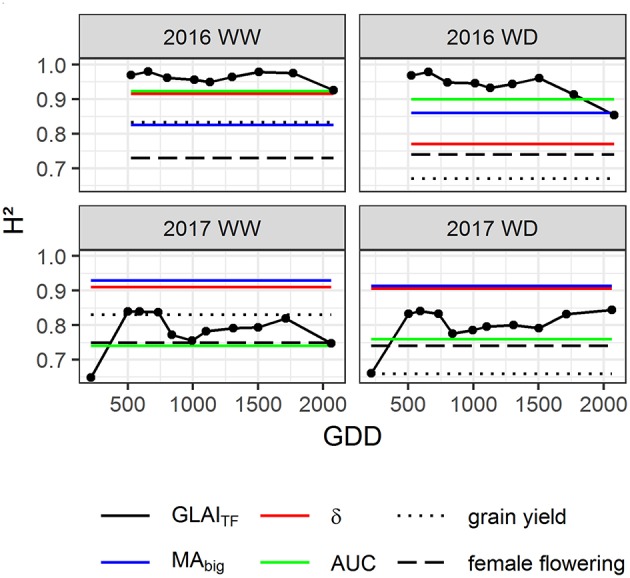
Heritability estimates for GLAI dynamics (*GLAI*_*TF*_), biggest leaf area (*MA*_*big*_), leaf longevity (δ), Area Under the Curve (*AUC*), grain yield and female flowering throughout the growth cycle in 2016 and 2017 for Well-Watered (WW) and Water-Deficient (WD) treatments. The black dots represent the heritability of *GLAI*_*TF*_ estimated at each flight date, year and modality.

To investigate the potential interest of GLAI traits (*MA*_*big*_, δ, *AUC*) in maize breeding, their heritability and their impact on grain yield and its components were evaluated. High heritability of *MA*_*big*_, δ and *AUC* traits was found for all years and conditions with *H*^2^ > 0.70 (Figure 6). This is consistent with the high heritability of *GLAI*_*TF*_ throughout the growth cycle. *GLAI*_*TF*_ heritability was slightly lower in 2017 than in 2016, probably because of density heterogeneities. Moreover, the lower heritability observed in 2017 for the first flight is explained by the dominant impact of density at this early stage, which had a medium heritability (0.53<H^2^<0.66). The senescence period also seems to show decreased heritability, which can be linked to the increasing residual effect of the soil reflectance, leading to poorer performance of the transfer function during this period (Girard and Girard, 2003). Moreover, the soil moisture heterogeneity may also contribute to decrease the heritability for the later stages, especially for the 2016 WD condition when the water stress is culminating. Overall, both the dynamics and the derived traits exhibit similar or higher heritability than grain yield and female flowering.

Earliness is related to the duration of the growth cycle, with early genotypes that tend to have fewer leaves, reduced stay-green, and finally lower grain yield (Li et al., 2016; Parent et al., 2018). In our study, the effect of GLAI traits on grain yield and its components are distinct from the effect of the flowering date which was considered in the linear model (Table 4). Further, grain yield and its components are more related to GLAI traits that to earliness. Among the four harvest traits (grain yield, kernel number, thousand kernel weight, harvest grain moisture), the grain yield is the best explained trait (Table 4). As it accounts for both the magnitude and the duration of the dynamics, *AUC* explains the largest part of the harvest traits variance, with up to 14.7 and 16.4%, for grain yield and kernel number, respectively. Cairns et al. (2012) and Christopher et al. (2014, 2016) also highlighted the link between grain yield and the AUC of NDVI dynamics in maize and wheat, respectively. Cairns et al. (2012) showed that AUC throughout the whole growth cycle explains up to 14% of grain yield variability under optimal conditions and 9% under drought stress in tropical hybrids, which agrees with our findings. However, a limited effect of *AUC* on grain yield and kernel number was observed in the 2016 WD condition, because of the greater impact of thousand kernel weight on grain yield due to water stress during grain filling (Çakir, 2004; Mangani et al., 2018).

**Table 4:** Impact of GLAI traits on grain yield and its components in each year and water treatment.

**Trial**		**Trait**	**r^**2**^** **adjusted**	**Flowering date**		***AUC***		**δ**		**MA**_*******big*******_	
				***p*-value**	**var. expl**.	***p*-value**	**var. expl**.	***p*-value**	**var. expl**.	***p*-value**	**var. expl**.
2016	WW	GY	0.40	***	0.111	***	0.147	***	0.025	n.s.	X
		KN	0.37	***	0.087	***	0.164	*	0.014	n.s.	X
		TKW	0.02	n.s.	X	n.s.	X	n.s.	X	***	0.031
		HGM	0.25	***	0.037	***	0.126	***	0.053	n.s.	X
	WD	GY	0.11	n.s.	X	***	0.030	**	0.020	**	0.025
		KN	0.05	n.s.	X	***	0.033	**	0.020	n.s.	X
		TKW	0.15	n.s.	X	n.s.	X	***	0.117	***	0.074
		HGM	0.32	***	0.048	***	0.065	***	0.130	*	0.009
2017	WW	GY	0.21	*	0.010	***	0.127	n.s.	X	–	–
		KN	0.17	n.s.	X	***	0.127	n.s.	X	–	–
		TKW	0.03	*	0.018	n.s.	X	n.s.	X	–	–
		HGM	0.04	n.s.	X	***	0.021	*	0.017	–	–
	WD	GY	0.12	n.s.	X	***	0.119	n.s.	X	–	–
		KN	0.15	n.s.	X	***	0.143	n.s.	X	–	–
		TKW	0	n.s.	X	n.s.	X	n.s.	X	–	–
		HGM	0.08	***	0.045	**	0.021	**	0.022	–	–

The effect of the flowering date, the Area Under the Curve (AUC), the leaf longevity (δ) and the area of the biggest leaf (MA_big_) on grain yield (GY) and its components (KN, kernel number; TKW, thousand kernel weight; HGM, harvest grain moisture) were tested and the effects size estimated. To prevent issues due to multicollinearity between predictors, MA_big_ was removed from the 2017 model based on the variance inflation factor. The significance of each effect is given by the “p-value” column with *** p ≤ 0.001; ** p ≤ 0.01; * p ≤ 0.05; n.s. p > 0.05. The magnitude of the effects is reported in the column “var. expl.” The adjusted r^2^ shows the total variance explained. The model was fitted on adjusted means.

*MA*_*big*_ and δ showed smaller effects, except for thousand kernel weight and harvest grain moisture in the 2016 WD condition. *MA*_*big*_ impact on grain yield and thousand kernel weight is consistent with results reported by Allison et al. (1998), Subedi and Ma (2005), and Li et al. (2018), showing the strong dependency of grain yield to the area of the biggest leaves around the ear. However, δ had a larger effect than *MA*_*big*_, due to the timing of the drought stress that impacted mostly the thousand kernel weight and harvest grain moisture through an earlier senescence and a shorter grain filling period (Çakir, 2004; Li et al., 2018; Mangani et al., 2018). Previous works (Cairns et al., 2012; Kante et al., 2016; Yang et al., 2017b) have also pointed out that stay-green is one of the major determinant of grain yield, and can explain from 7 to 12% of its variation, which is very similar to the magnitude of δ effect in this study (Table 4). Finally, it is noteworthy that *MA*_*big*_ and δ are strongly negatively correlated in both stressed and well-watered environments (Supplementary Figure 4). This relationship, also observed by Kamara et al. (2003), might be the consequence of an increase illumination homogeneity within canopy strata for maize genotypes with smaller leaves, thus exhibiting a delayed senescence.

Leaf greenness traits have been shown to increase breeding efficiency when used as secondary traits in the past (Bänziger and Lafitte, 1997; Rutkoski et al., 2016; Sun et al., 2017). There are several requirements for a secondary trait to be useful in a breeding program: it must be correlated with yield, have a higher heritability than yield, be fast, easy and cheap to measure by non-destructive means (Araus et al., 2012). The use of the GLAI phenotyping method proposed in this study fulfill all these requirements. Importantly, it provides GLAI traits that exhibit similar or higher heritability than their ground based counterparts. The heritability of *MA*_*big*_ is comparable with that of maize ear leaf width and length measured manually (Tian et al., 2011; Wang et al., 2017, 2018; Zhao et al., 2019), while stay-green traits, related to δ, are generally associated with lower heritability, whether assessed manually (Yang et al., 2017b), by visual scoring (Messmer et al., 2011; Ziyomo and Bernardo, 2013; Almeida et al., 2014; Trachsel et al., 2016) or proximal sensing (Christopher et al., 2014; Yang et al., 2017b). Similarly, lower heritability is generally reported for traits similar to *AUC*, such as the NDVI AUC before flowering (Trachsel et al., 2016 for maize) and after flowering (Christopher et al., 2014 for wheat) estimated by proximal sensing. These results demonstrate the benefit of data aggregation at the canopy level throughout the whole growth cycle, by combining a model-based approach with UAV remote sensing to characterize traits describing the green leaf area dynamics. These spatial and temporal aggregation decreases estimation uncertainties compared to measurements realized on few plants and/or a single time point, and provides more accurate genotypic parameters (Araus et al., 2012; Tuberosa, 2012). These conclusions are in good agreement with a recent study that demonstrated in durum wheat the higher heritability of NDVI estimated from UAV compared to ground-based NDVI (Condorelli et al., 2018).

Our results demonstrate that *AUC*, *MA*_*big*_ and δ are promising traits for further investigation in maize breeding programs. However, the compensation between leaf area (*MA*_*big*_) and leaf longevity (δ), constitutes a significant limit for the improvement of maize through increased light interception during the whole cycle and particularly the grain filling period. A solution to circumvent this correlation could be to modify maize leaf area vertical profile and leaf orientation to allow a better light penetration in the canopy, but this question can't be addressed with the simple GLAI dynamics model presented here.

### Applicability and Limitations of the Method

In this study, we proposed a high-throughput method to phenotype maize GLAI in the field by combining repeated UAV observations, a simple GLAI dynamics model and a few field measurements. The main limitation of the proposed method is that two parameters, the phyllochron (ϕ_*GDD*_) and the final number of leaves (*i*_*top*_), must be characterized beforehand for all the genotypes. These two parameters were assumed constant under contrasted environmental conditions to reduce the field work. The leaf number is marginally sensitive to growing conditions, with maximum variation of about one leaf (Allen et al., 1973; Bonaparte and Brawn, 1976). Although the constancy of the phyllochron across environments for a given genotype is a common assumption in crop modeling (Tardieu, 2013), it is still debated. For example, Birch et al. (1998) for maize and Clerget et al. (2008) for sorghum found that phyllochron may vary between environment due to temperature, day length and irradiance variation. Conversely, Lafarge and Tardieu (2002) measured maize phyllochron in strongly diverse environments located in France (Mediterranean conditions) and in Mali (Sahelian conditions) and showed that maize phyllochron was perfectly stable between environments and years except under extreme conditions of temperature (40°C) and high vapor pressure deficit (6 kPa). Chenu et al. (2008) showed the same constancy for sorghum phyllochron across environments. Therefore, the phyllochron and leaf number constancy for a given genotype seems to be a reasonable assumption for most of agricultural environments in which our method is likely to be used.

The phyllochron and the leaf number can be assessed rapidly either in the field or in a high-throughput greenhouse platform during the early stages before silking (Tardieu et al., 2017). This first step provides an opportunity to gather information on the vegetative development of a population of interest and is necessary to accelerate the characterization of its GLAI dynamics under a subsequently infinite number of scenarios. The third prior variable used, the density, is often routinely measured in a breeding trial. If the density is unknown, it could be readily estimated through image analysis based on early UAV flight as proposed by Gnädinger and Schmidhalter (2017) and Jin et al. (2017).

The preliminary characterization of the phyllochron and the leaf number should be done once but for all new genotypes, and thus could not be feasible for some material, like segregating breeding populations. However, in case one or all the prior variables are unknown, it is still possible to assess GLAI dynamics with acceptable precision. Indeed, we showed that using only the multispectral measurements lead to reasonable GLAI estimation performance throughout the cycle. Indeed, spectral predictors limit the RRMSEP inflation during the senescence which is the period exhibiting the higher uncertainties. Moreover, a highly precise GLAI dynamics is only required to estimate *MA*_*big*_ and δ when inverting the GDMM. Indeed, we demonstrated that *GLAI*_*est*_ was accurately retrieved irrespectively to the use of the prior variables, and thus emerging properties such as AUC can still be derived accurately. This alternative approach is thus particularly well suited for the first steps of breeding programs, when the need is on high-throughput more than high precision phenotyping tools.

## Conclusion

This study demonstrated that with a limited number of field measurements and UAV multispectral observations covering the growth cycle, it is possible to finely characterize the GLAI dynamics of a large maize panel under contrasted environmental conditions. Our high-throughput method reduces the phenotyping time by a factor of about 20 compared to fully ground-based phenotyping observations. Moreover, the use of a simple GLAI dynamics model provides continuous description from emergence to maturity and allows the estimation of three GLAI traits: the Area Under the Curve, the area of the biggest leaf and leaf longevity. Both the dynamics and the GLAI traits exhibit high heritability and could be used as secondary traits in maize breeding programs. Indeed, the GLAI traits can be used to predict grain yield while the pattern of GLAI dynamics drought response is informative about the grain yield stability under water stress. Finally, the high-throughput nature of the method proposed in this study also paves the way to new genetic studies on large populations, like Genome Wide Association Studies, to dissect the genetic determinants of GLAI and its interaction with the environment throughout the crop growth cycle.

## Author Contributions

JB, FB, SP, and AC designed the experiment. DD managed the UAV workflow. The analysis and interpretation were mainly accomplished by JB, with the help of M-HT, MW, and FB. JB wrote the manuscript with significant revisions from FB.

### Conflict of Interest Statement

The authors declare that the research was conducted in the absence of any commercial or financial relationships that could be construed as a potential conflict of interest.

## Abbreviations

AUC: Area Under the Curve
GDMM: GLAI Dynamics Maize Model
GLAI: Green Leaf Area Index
NDVI: Normalized Difference Vegetation Index
RMSE: Root Mean Squared Error
RMSEP: Root Mean Squared Error of Prediction
RRMSE: Relative Root Mean Squared Error
RRMSEP: Relative Root Mean Squared Error of Prediction
UAV: Unmanned Aerial Vehicle
WD: Water-Deficient
WW: Well-Watered.
