# Supplementary material for: A High-Throughput Model-Assisted Method for Phenotyping Maize Green Leaf Area Index Dynamics Using Unmanned Aerial Vehicle Imagery
Source: Front Plant Sci. 2019 Jun 6;10:685. doi: 10.3389/fpls.2019.00685 (PMC6568052; doi:10.3389/fpls.2019.00685)
Supplement: Supplementary file 1 [file Presentation_1.pdf]

Supplementary Material

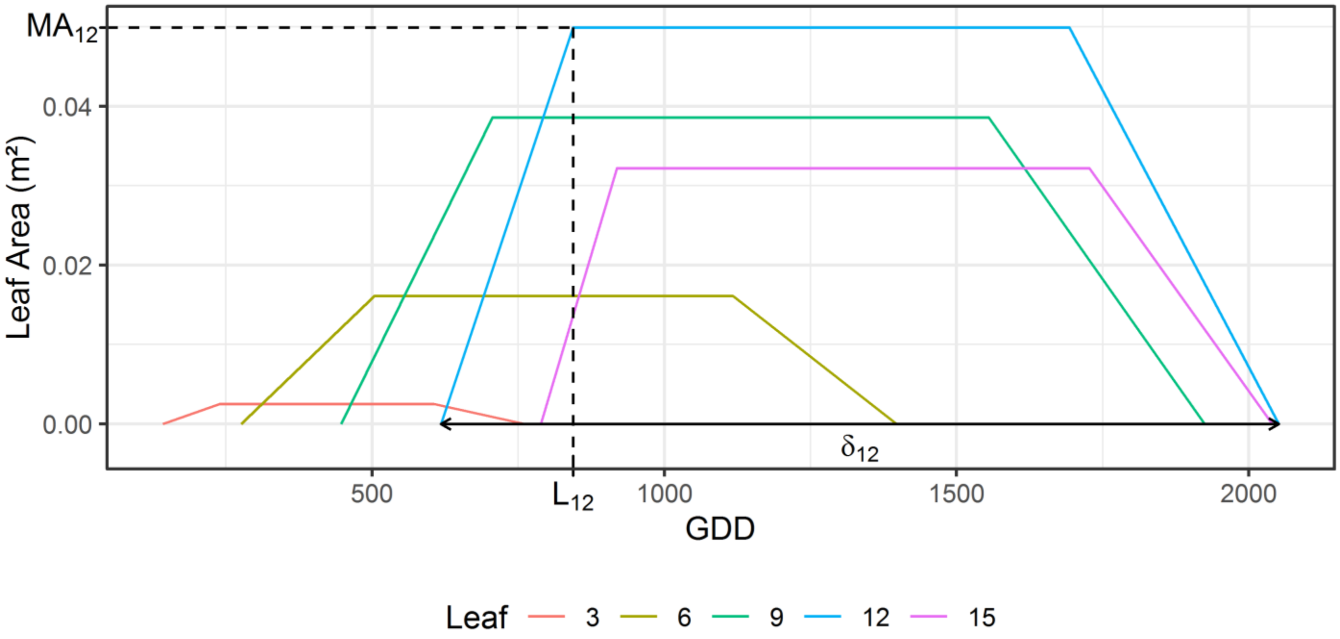

Supplementary Figure 1. Example of the evolution of the area of five leaves throughout the growth cycle in the GLAI Dynamics Maize Model (GDMM). For leaf number 12, the time of the ligule appearance (L_{12}), maximum area (MA_{12}) and longevity (δ_{12}) are also reported.

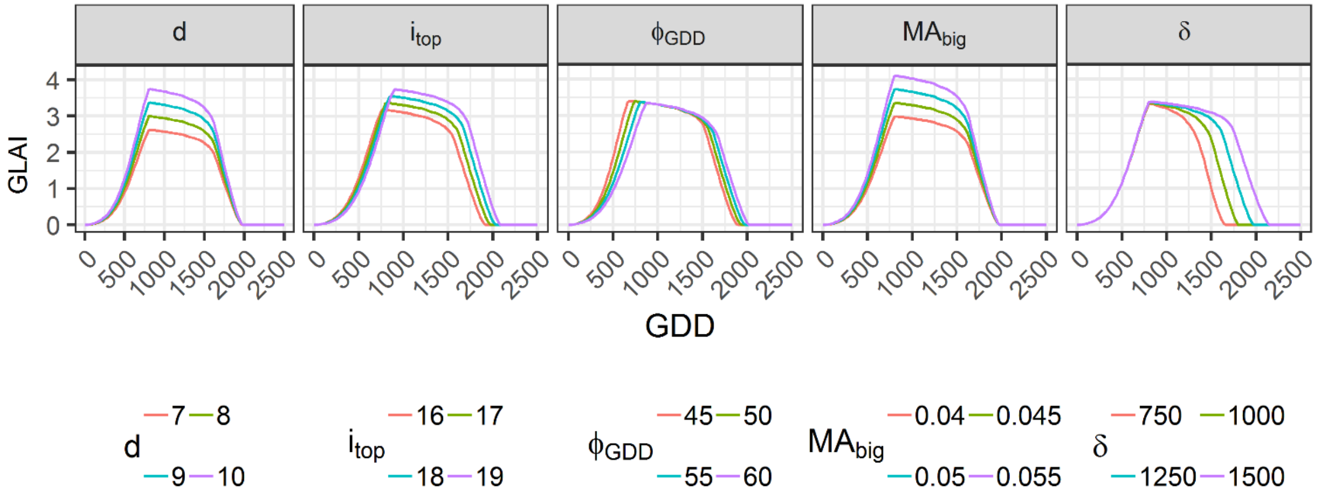

Supplementary Figure 2. Sensitivity analysis of the GLAI Dynamics Maize Model (GDMM). Changes in GLAI over thermal time (GDD) are presented for different values of density (d), final leaf number (i_{top}), phyllochron (ϕ_{GDD}), biggest leaf area (MA_{big}) and leaf longevity factor (δ).

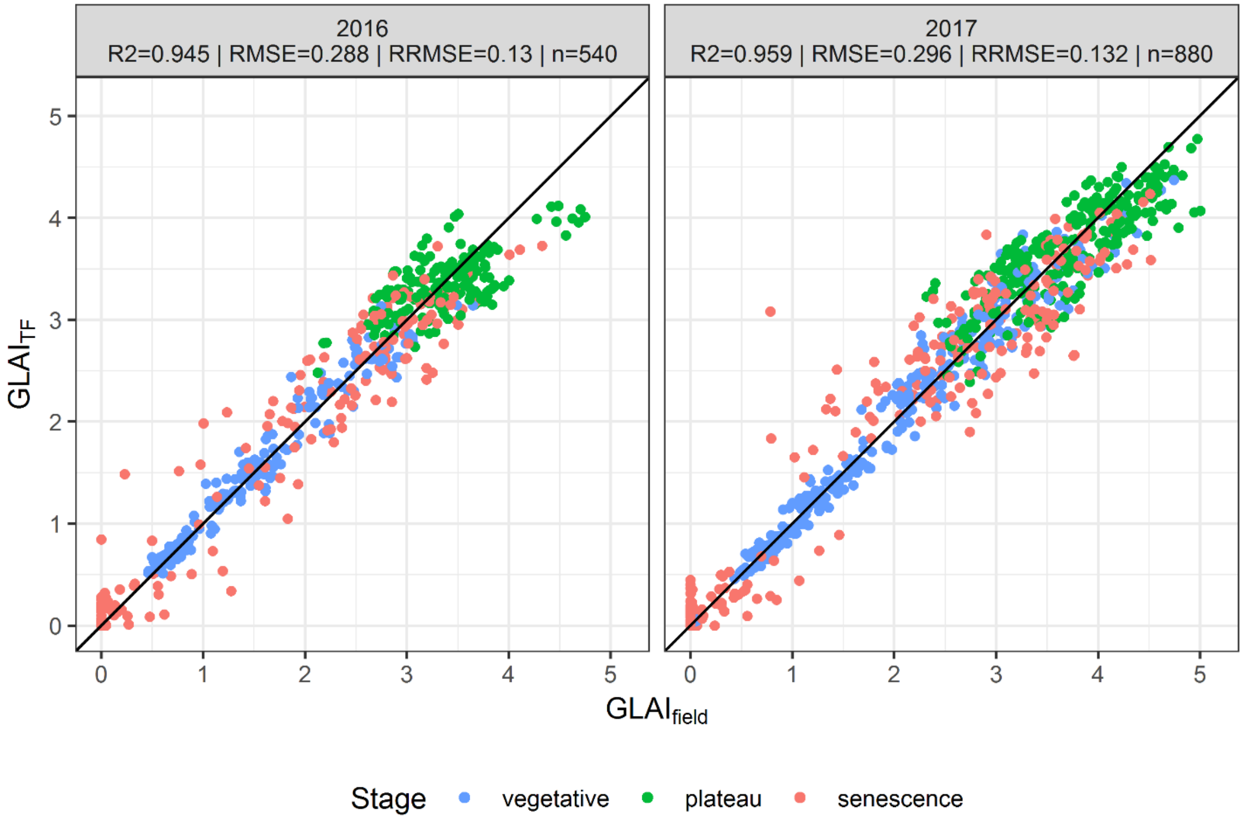

Supplementary Figure 3. Relationship between the GLAI predicted with the transfer functions ($GLAI_{TF}$) and estimated from field measurements ($GLAI_{field}$) in 2016 and 2017. One dot represents one reference microplot at one of the UAV flight dates.

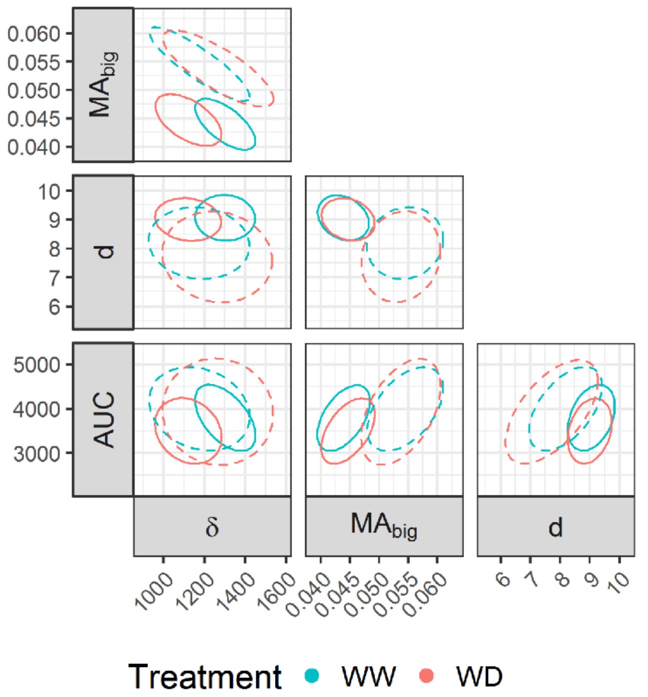

Supplementary Figure 4. Joint distributions of the Area Under the Curve (AUC), leaf longevity (δ), biggest leaf area (MA_{big}) and density (d) over the two years and the two water treatments. Distribution of traits measured in 2016 are represented with continuous lines, while 2017 data are represented with dashed lines. Each ellipse represents 95% of the data. Data are adjusted means.

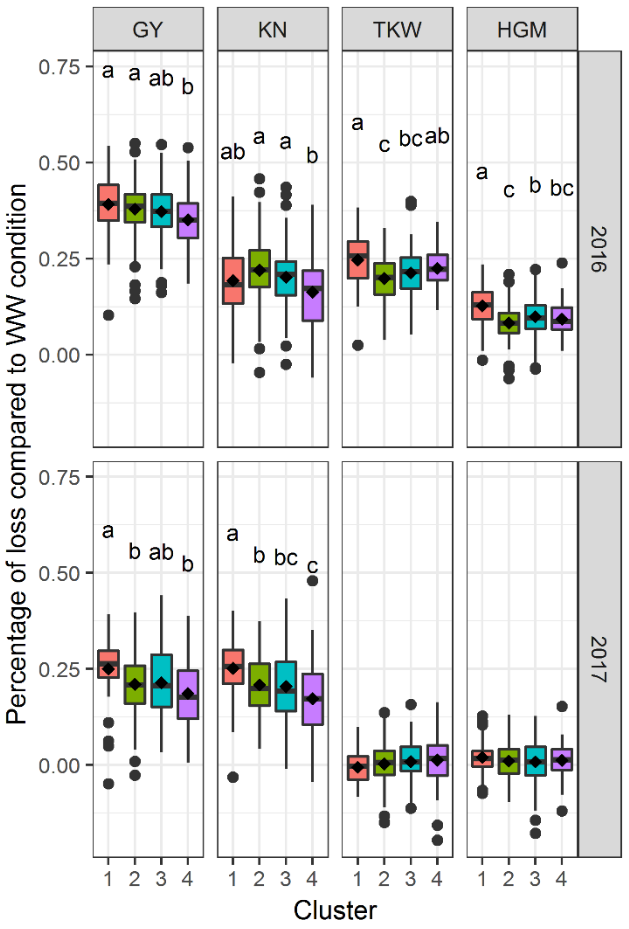

Supplementary Figure 5. Boxplots of Water-Deficient (WD) losses compared to Well-Watered condition (WW) for grain yield (GY), kernel number (KN), thousand kernel weight (TKW) and harvest grain moisture (HGM) in 2016 and 2017. The horizontal line in the boxplots corresponds to the median, while the diamond corresponds to the mean. The lower and upper hinges show the first and third quartiles, and the whiskers correspond to 1.5 times the inter-quartile range or to the most extreme value, whichever is smallest. Dots represents values outside this range. Letters are featured above the boxplots if the cluster effect is significant. Different letters indicate a significant difference between clusters based on post-hoc Tukey tests (α=0.05). No letters are visible when the effect of the clusters on the trait considered is not significant. The linear model was fitted on the normalized difference of adjusted means between the WW and WD conditions.
